# *Thymus richardii* subsp. *nitidus* (Guss.) Jalas Essential Oil: An Ally against Oral Pathogens and Mouth Health

**DOI:** 10.3390/molecules28124803

**Published:** 2023-06-16

**Authors:** Giusy Castagliuolo, Michela Di Napoli, Alessandro Vaglica, Natale Badalamenti, Dario Antonini, Mario Varcamonti, Maurizio Bruno, Anna Zanfardino, Giuseppe Bazan

**Affiliations:** 1 Department of Biology, University of Naples Federico II, 80126 Naples, Italy; giusy.castagliuolo@unina.it (G.C.); michela.dinapoli@unina.it (M.D.N.); dario.antonini@unina.it (D.A.); varcamon@unina.it (M.V.); 2 Department of Biological, Chemical and Pharmaceutical Sciences and Technologies (STEBICEF), Università degli Studi di Palermo, Viale delle Scienze, ed. 17, 90128 Palermo, Italy; alessandro.vaglica@unipa.it (A.V.); natale.badalamenti@unipa.it (N.B.); maurizio.bruno@unipa.it (M.B.); giuseppe.bazan@unipa.it (G.B.); 3 NBFC—National Biodiversity Future Center, 90133 Palermo, Italy

**Keywords:** *Thymus richardii* subsp. *nitidus*, Lamiaceae, essential oil, *β*-bisabolene, thymol, antimicrobial, oral pathogens, antibiofilm and antioxidant properties

## Abstract

The genus *Thymus* L., belonging to the Lamiaceae family, contains about 220 species with a distribution that mainly extends in Europe, northwest Africa, Ethiopia, Asia, and southern Greenland. Due to their excellent biological properties, fresh and/or dried leaves and aerial parts of several *Thymus* ssp. have been utilized in the traditional medicine of many countries. To evaluate not only the chemical aspects but also the biological properties, the essential oils (EOs), obtained from the pre-flowering and flowering aerial parts of *Thymus richardii* subsp. *nitidus* (Guss.) Jalas, endemic to Marettimo Island (Sicily, Italy), were investigated. The chemical composition of the EOs, obtained by classical hydrodistillation and GC-MS and GC-FID analyses, showed the occurrence of similar amounts of monoterpene hydrocarbons, oxygenated monoterpenes, and sesquiterpene hydrocarbons. The main constituents of the pre-flowering oil were *β-*bisabolene (28.54%), *p*-cymene (24.45%), and thymol methyl ether (15.90%). The EO obtained from the flowering aerial parts showed as principal metabolites *β-*bisabolene (17.91%), thymol (16.26%), and limonene (15.59%). The EO of the flowering aerial parts, and its main pure constituents, *β-*bisabolene, thymol, limonene, *p*-cymene, and thymol methyl ether were investigated for their antimicrobial activity against oral pathogens and for their antibiofilm and antioxidant properties.

## 1. Introduction

The genus *Thymus* L. (Lamiaceae) is considered one of the largest genus in the Lamiaceae family, comprising approximately 220 accepted species. Most of these species are chamaephytes, and they are distributed throughout Europe, northwest Africa, Ethiopia, Asia, and southern Greenland [1,2]. Due to its pleasant flavor and nutritional and medicinal values, *Thymus* ssp. has been largely employed in the food, pharmaceutical, cosmetic, and perfume industries [3,4]. Due to their excellent biological properties, the fresh or dried leaves and flowering parts of several *Thymus* ssp. have been utilized in the traditional medicine of many countries as antimicrobial, anti-spasmodic, and antioxidant treatments for different digestive and respiratory illnesses [5].

Several *Thymus* ssp. extracts have been investigated for their non-volatile organic compounds. The main metabolites occurring in them are flavonoids, phenylpropanoids, lignans, tannins, organic acids, and terpenoids. Furthermore, the antimicrobial, antioxidant, antitumor, anti-inflammatory, analgesic, antispasmodic, carminative, anti-hypertensive, anti-diabetic, etc., properties of many of them have been proven both in vitro and in vivo [5]. *Thymus* plants, such as *T. vulgaris* L., *T. serpyllum* L., *T. pulegioides* L., and *T. zygis* L., have a rich historical background and hold significant commercial value [6]. Traditionally, these plants have been utilized in various ways, such as tea, spices, and traditional remedies, due to their favorable effects on digestive and respiratory ailments [4,7]. *Thymus* plants are considered warming and pungent in traditional Chinese medicine, stimulating appetite and aiding digestion [8]. Furthermore, a comprehensive review has been conducted on the phytochemical and pharmacological investigations of *T. daenensis* Celak from Iran, used extensively in folk medicine [9]. In fact, the medicinal properties of *Thymus* plants are widely acknowledged, including their use for inflammatory conditions, cardiovascular diseases, kidney disorders, and women’s health concerns [10,11,12].

For instance, in Spain, *T. vulgaris* is used for postpartum cleansing [13]. In India, *T. serpyllum* is recognized for its effectiveness in treating menstrual disorders [14]. In Tunisia, *T. algeriensis* Boiss. and Reut. is believed to prevent abortion and complications during pregnancy [15]. Apart from their medicinal properties, *Thymus* plants are extensively utilized in culinary preparations for their aromatic qualities and taste. They are incorporated into various recipes, including baked goods, sauces, meats, vegetarian dishes, desserts, and fresh salads [16,17]. In Italy, dried *Thymus* leaves are also combined with other herbs for perfuming clothes or rooms [14].

Furthermore, most of the published papers concerning the essential oils (EOs) of *Thymus* ssp. were characterized, in many cases, by the occurrence of two main aromatic compounds, carvacrol and thymol, together with a minor quantity of *p*-cymene and *γ*-terpinene [18,19]. Other metabolites present in the EOs are linalool, borneol, and 1,8-cineole [20,21].

Furthermore, thanks to their antimicrobial and/or antioxidant compounds, EOs of the *Thymus* species have been used in recent years as alternatives to commercial synthetic chemicals. In fact, to prolong the shelf-life of fresh foods, they have been incorporated into packaging materials [22,23,24], used as corrosion inhibitors for different metals in various acids [25], and applied in the disinfection of historical art materials [26,27,28,29].

Based on current knowledge, there are 20 *taxa* present in Italy [30], of which five are found in Sicily. The Sicilian *taxa* consist of *T. spinulosus* Ten. and *T. paronychioides* Čelak., which belong to *Thymus* sect. *Hypodromi*, as well as *T. richardii* subsp. *nitidus* (Guss.) Jalas, *T. longicaulis* C. Presl, and *T. praecox* subsp. *parvulus* (Lojac.) Bartolucci, Peruzzi, and N.G.Passal, which belong to Th. sect. *Serpyllum* [31].

*Thymus richardii* s.l., according to Euro + Med PlantBase [32], has a distribution that extends beyond Sicily, Spain, and the Balkans. Within the species, four subspecies are distinguished: *T. richardii* Pers. subsp. *richardii* [syn. *T. aureopunctatus* (Beck) K. Malý] present in the Balkans and Baleares Islands; *T. richardii* subsp. *ebusitanus* (Font Quer) Jalas [syn. *T. ebusitanus* (Font Quer) Romo] exclusive to the Baleares Islands; *T. richardii* subsp. *vigoi* Riera, Güemes and Rosselló growing in Spain; and *T. richardii* subsp. *nitidus* (Guss.) Jalas [syn. *T. nitidus* Guss.; *T. lucidus* Guss.] endemic of the island of Marettimo (Sicily, Italy).

**Figure 1:**
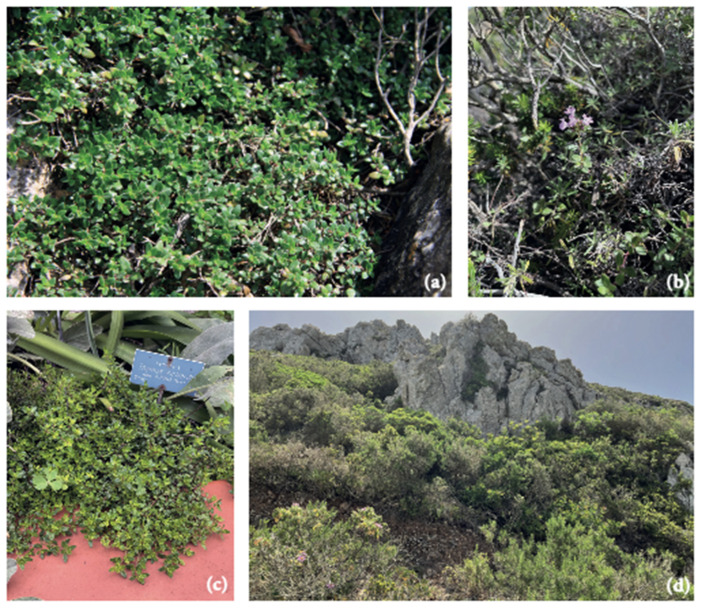
The habitus of *Thymus richardii* subsp. *nitidus* (**a**); inflorescence with purplish flowers (**b**); *T. richardii* subsp. *nitidus* in the collection of Palermo Botanical Garden (**c**); the rocky habitats at Punta Madonnuzza on Marettimo Island (**d**).

*Thymus richardii* subsp. *nitidus* (Guss.) Jalas, [≡ *Thymus nitidus* Guss. ≡ *Thymus serpyllum* var. *nitidus* (Guss.) Bég.] (Figure 1) is a chamaephyte that grows 8–15 cm tall. It has woody, ascending, or suberect stems with amphitrichous indumentum (hairs on two opposite sides).

Its lanceolate leaves are 7–9 mm long and 3–4 mm wide, glabrous, and not ciliate on the margin. The subspherical inflorescence has purplish flowers, and the calyx is hirsute with glandular hairs, while the corolla is 7–9 mm long. Flowering occurs from May to June [33]. Morales [34] reported that the chromosome number for this plant is 2*n* = 28. This *taxon* is endemic to Marettimo Island (W. Sicily). It grows on habitats with rocky, calcareous substrates and occurs in five localities on the island: Mt. Lissandro, Semaforo, Punta Anzini, Libbano, and Punta Madonnuzza (200–600 m a.s.l.) [35,36].

*Thymus richardii* subsp. *nitidus* can be considered one of the rarest thyme species in Italy, although it is listed as Near Threatened (NT) [37]. This is because its habitat is not significantly threatened and the population is mostly stable [38].

The only previous report on the biological properties of *T. richardii* subsp. *nitidus* concerns the methanolic extract of the aerial parts that was screened for its inhibitory effect on the production of leukotriene B4 by 5-lipoxygenase in intact cells. It showed remarkable activity, inhibiting almost completely the LTB4 production in intact rat PMNL at 200 μg/mL. This effect was maintained even at a dose of 50 μg/mL, indicating its possible use as a source of potent 5-LOX inhibitors [39].

Essential oils are effective antioxidants, mostly because of their activity in food preservation [40], and they are known to possess anti-carcinogenic, antimicrobial, and anti-inflammatory properties due to over 200 constituents [41,42]. Essential oils are a mixture of volatile constituents produced by aromatic plants, serving as a protective mechanism against microorganisms [43]. Tea tree, thyme, cinnamon, citrus, bergamot, lavender, peppermint, and many other EOs were used in dentistry to counteract bacterial pathogen action [44]. To increase antibiotic resistance and for economic reasons, people still use natural products for primary healthcare [45].

Consequently, as a continuation of our research on plants of the Mediterranean area [46,47,48,49] and their EOs biological properties [50,51,52], in the present study, it is described the EO composition of the aerial parts of *T. richardii* subsp. *nitidus*, collected at two different vegetative stages, as well as the biological properties of the EO obtained from the full-flowering aerial parts. In addition, it is reported the antimicrobial, antibiofilm, and antioxidant effects of the EO of the flowering aerial parts and of its main pure constituents, *β-*bisabolene and thymol.

## 2. Results and Discussion

### 2.1. Chemical Composition of the Essential Oils

Hydrodistillation of *T. richardii* subsp. *nitidus* aerial parts collected at a pre-flowering stage (**PF**) gave a yellow EO. Overall, sixteen compounds were identified, representing 98.13% of total components, listed in Table 1 according to their retention indices on a DB-Wax column and classified into four classes based on their chemical structures. Monoterpene hydrocarbons formed the main class, representing 33.92% of the total, with *p*-cymene (24.45%) and limonene (6.23%) as the most abundant components. Sesquiterpene hydrocarbons occurred in similar amounts (33.76%), with *β*-bisabolene (28.54%) being the principal constituent of the class and of the EO. Oxygenated monoterpenes were also present in large amounts (30.17%), with thymol methyl ether (15.90%), thymol (4.56%), and carvacrol (4.49%) as the main components of this class.

The EO obtained from the aerial parts collected at the full flowering stage (**F**) showed a similar chemical profile. In this case, twenty-one metabolites were identified, representing 96.72% of the total composition. In this case, the main class was represented by oxygenated monoterpenes (39.65%), showing a larger amount of thymol (16.26%) and carvacrol (7.82%) with respect to **PF** and a minor quantity of thymol methyl ether (8.87%). It is noteworthy for the presence of linalool (6.70%), which is practically absent in **PF** (0.18%). The main constituent of the EO was always *β*-bisabolene (17.91%), but it was present in a minor amount with respect to **PF**. Among the sesquiterpene hydrocarbons, it must also be mentioned the good occurrence of germacrene D (6.14%). In **F**, among monoterpene hydrocarbons (29.27%), limonene (15.59%) represented the main metabolite, while *p*-cymene occurred only for 9.04%.

In a previous report [53], the EO of *Thymus richardii* subsp. *nitidus*, always collected on Marettimo Island but at a post-flowering stage, was analyzed. Thirty-six compounds were identified, among which *β*-bisabolene (32.30%), carvacrol (13.10%), thymol methyl ether (12.40%), *trans*-dihydrocarvone (5.l0%), *t*-cadinol (4.00%), and *β*-caryophyllene (3.40%) were identified as the main constituents. Successively, Llorens et al. [54] investigated the chemical compositions of the EOs of several *Thymus* taxa belonging to *T. richardii* Pers., namely *T. richardii* subsp. *richardii* from Bosnia and Majorca (Spain), *T. richardii* subsp. *ebusitanus* (Font Quer) Jalas from Ibiza (Spain), *T. richardii* subsp. *vigoi* Riera, Güemes and Rosselló from Valencia (Spain), as well as *T. richardii* subsp. *nitidus* from Marettimo (Sicily, Italy). In this investigation, the main constituents of the EO of *T. richardii* subsp. *nitidus*, collected at the full flowering stage, were *p*-cymene (25.10–15.30%), *β*-bisabolene (17.70–16.60%), limonene (16.70–8.80%), thymol methyl ether (14.90–2.40%), carvacrol (15.20–0%), and thymol (13.90–1.50%). Our results are quite similar to the previously reported investigations; in fact, *p*-cymene (24.45% and 9.04%, for **PF** and **F**, respectively), *β*-bisabolene (28.54% and 17.91%, for **PF** and **F**, respectively), limonene (6.23% and 15.59%, for **PF** and **F**, respectively), thymol methyl ether (15.90% and 8.87%, for **PF** and **F**, respectively), thymol (4.56% and 16.24%, for **PF** and **F**, respectively), and carvacrol (4.49% and 7.82%, for **PF** and **F**, respectively) also occurred in good amounts.

From the comparison with the chemical compositions of EOs from other species of the *Thymus* genus belonging to the same section (*Serpyllum*) and subsection (*Insulares*), a clear difference in the composition of **F** was observed.

Among the main compounds obtained in this study, there was only the common presence of thymol in a similar percentage (20%) with *T. dreatensis* Bratt, whose EO was found to have the ability to remove hydroxyl radicals and prevent the degradation of deoxyribose [55], while with *T. guyonii* de Noè, that showed antioxidant activity, the presence of *p*-cymene (19%), thymol (11%), and thymol methyl ether (11%) was observed [56]. On the other hand, the composition of the EO of *T. willkommii* Ronniger was found to be completely different, with *α*-terpenyl acetate (36–69%) and linalool (0–57%) as the main secondary metabolites [20]. As the main constituent, *β*-bisabolene was not found in other plants of the *Thymus* genus except for another one belonging to the same section but a different subsection (*Alternantes*), namely *T. pulegioides* L. subsp. *similialpestris* Debray, but in a lesser amount (7%) [57]. As regards *Thymus vulgaris* EOs, these have been shown to have important antibacterial and anti-biofilm activity, exhibited in seven distinct chemotypes characterized by chemical variability for the presence of compounds like thymol, linalool, carvacrol, geraniol, thujanol-4, terpineol, and 1,8-cineole [58,59]. In these compositions, the only main metabolites in common were thymol, carvacrol, and linalool, which, however, were present in greater abundance than those of *T. richardii* subsp. *nitidus*.

### 2.2. Antimicrobial Activity of T. richardii EO (**F**)

The *Thymus* genus exhibited potent antimicrobial activity against a wide range of microorganisms, and its primary bioactive components were EOs, particularly thymol [3,60]. These EOs have demonstrated significant inhibitory effects on both susceptible and resistant bacterial strains, and they have also exhibited strong synergistic effects when combined with other antimicrobial drugs, such as norfloxacin, clotrimazole, nystatin, and ketoconazole [61,62,63]. For example, the EO fraction of *T. magnus* (Nakai) Nakai, along with its major constituents, effectively inhibited *Salmonella typhimurium*, *Staphylococcus aureus*, and *Streptococcus pneumoniae* strains, with minimum inhibitory concentrations (MICs) ranging from 0.125 to 8 mg/mL. Notably, a synergistic effect was observed when combined with norfloxacin against *S. aureus* strains [62]. *Thymus capitatus* (L.) Hoffmanns and Link EOs, incorporated into phospholipid vesicles, demonstrated efficacy against oral cavity bacteria, including cariogenic *Lactobacillus acidophilus*, *Streptococcus mutans*, and commensal *Streptococcus sanguinis*, suggesting their potential in oral cavity disease treatment. The primary metabolite found in *T. capitatus* EO was carvacrol, present at a concentration of approximately 817 mg/mL [64]. *Thymus vulgaris* EO exhibited bacteriostatic activity against two major foodborne pathogens, *Listeria monocytogenes* and *S. aureus*, thanks to its high levels of *p*-cymene (47.9%) and thymol (43.1%) [65].

*Thymus* EO (**F**) extracted from fully flowering plants was tested on Gram-positive and Gram-negative bacteria by a modified Kirby and Bauer assay. Since there is an inhibition halo, the bacterial growth decreases when the quantity of EO increases.

**Figure 2:**
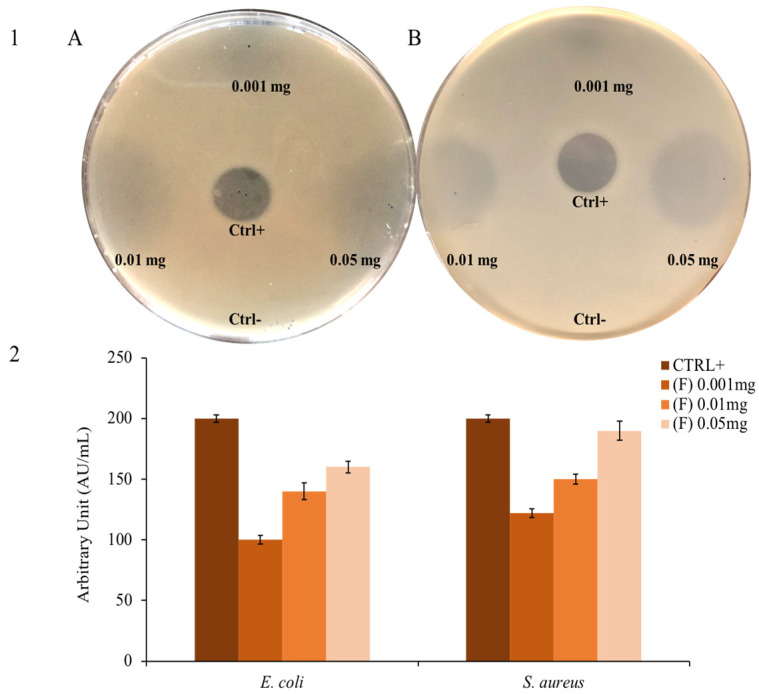
Bacterial growth inhibition: panel (**1**) shows the inhibition halo of (**F**) against (**A**) *E. coli* and (**B**) *S. aureus.* The positive control is ampicillin; panel (**2**) shows the inhibition halo expressed in AU/mL.

Figure 2 shows the inhibition halo formed by the antibiotic (positive control) and the absence of the DMSO halo in which the EO is resuspended (negative control). As also shown in Figure 2, the EO appears to be active on both Gram-negative *E. coli* and Gram-positive *S. aureus* model strains. This type of analysis is commonly used as a first approach, which represents a qualitative screening to understand if an EO or a compound has antimicrobial activity [66].

**Figure 3:**
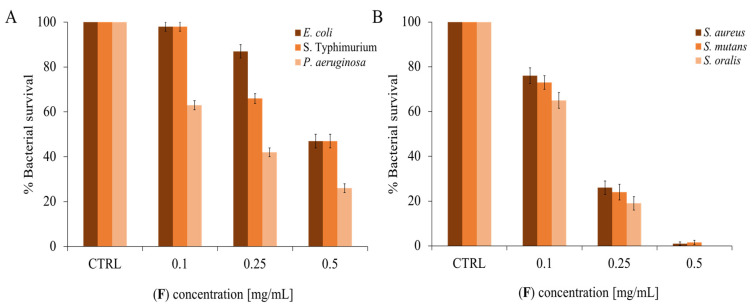
(**F**) Antimicrobial activity against panel (**A**): Gram-negative strains; panel (**B**)*:* Gram-positive strains. The assays were performed in three independent experiments. Standard deviations are always less than 10%.

To deepen the analysis of the antimicrobial activity, dose-response curves were carried out, increasing the concentration of EO and evaluating the survival of different bacteria. Dental researchers are developing and testing new therapeutic substances that are low- or non-toxic to prevent or eradicate dental plaque-related disorders. For this reason, three oral pathogenic strains were chosen as Gram-positive (*S. mutans*, *S. oralis*, and *S. aureus*) [67] and three oral and/or opportunistic pathogenic strains as Gram-negative bacteria (*P. aeruginosa*, *S*. Typhimurium, and *E. coli*) [68]. As can be seen in Figure 3, there is a proportionality between the increase in EO concentration and the decrease in bacterial survival. In general, the EO appears to be active at lower concentrations on Gram-positive bacteria than on negative ones.

A study of several *Thymus* species by Ballester-Costa et al. [69] suggests that organic EOs of *T. mastichina* L., *T. zygis*, *T. capitatus*, and *T. vulgaris* could be used as antibacterial agents in food preservation. These EOs can be accepted by consumers and authorized by regulatory agencies as natural preservative agents in organic foods.

Recent studies highlight the ability of *Thymus* to produce not only EOs but also methanolic extracts and volatile substances that have good antimicrobial activity. According to a study conducted by Vassiliou and collaborators [70], the EOs can be used in conjunction with conventional antibiotics. This approach may permit a reduction in the concentration of the synthetic antibiotic, in side effects, and in antibiotic resistance too. This strategy has been used for many years now with clavulanic acid and amoxicillin, for example.

Based on several studies and considering some EOs possible applications, it was decided to conduct further analysis on the antimicrobial effect of this interesting EO of *T. richardii* subsp. *nitidus* species.

To complete the analysis of the antimicrobial activity and make it quantitative, three independent experiments were performed to determine the MIC values through the microdilution method. As shown in Table 2, the lowest MIC values are observed against Gram-positive bacteria, with *S. oralis* CECT 8313 being the most sensitive. The MIC values found with (**F**) are very interesting because they are lower than most of the other EOs [71].

### 2.3. Antimicrobial Activity of the Components Present in T. richardii EO (F)

**Figure 4:**
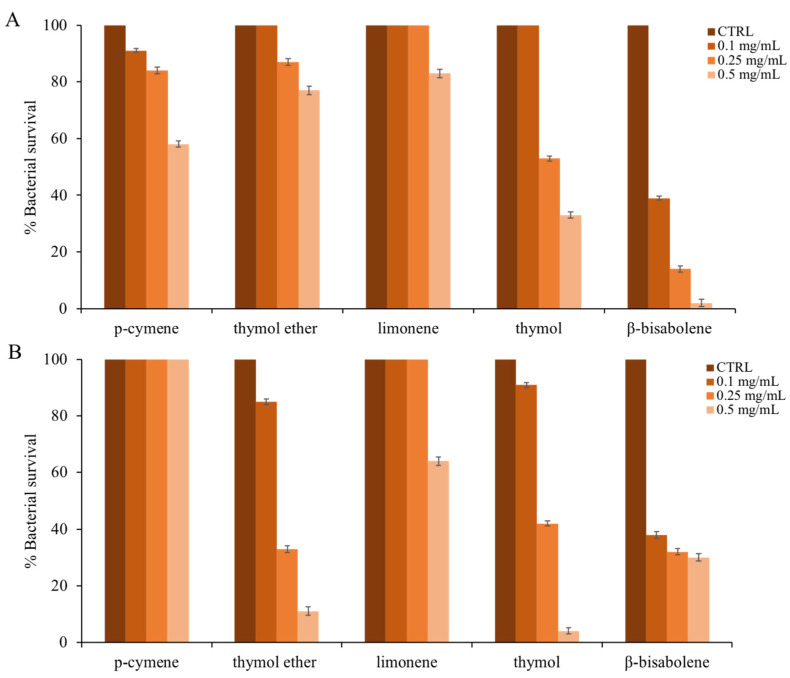
Antimicrobial activity of the main components presented in (**F**) against panel (**A**): *E. coli*; panel (**B**): *S. aureus.* The assays were performed in three independent experiments. Standard deviations are always less than 10%.

They have analyzed the antimicrobial activity of the single main compounds, which is more representative in terms of percentage amount within the EO of *T. richardii* (indicated in bold in the last column of Table 1). In order, *p*-cymene, thymol ether, limonene, thymol, and *β-*bisabolene were tested. Figure 4 shows the proportionality between the increase in compound concentration and the decrease in cell survival against *E. coli* (panel A) and *S. aureus* (panel B).

The single compounds were used at different concentrations, which respected the quantities present in the EO at 0.5 mg/mL.

The effect of each single compound seems more directed against the Gram-positive bacterial model (thymol ether, thymol, and *β-*bisabolene). However, the exception is *p*-cymene, which works (at the used concentrations) only on the *Escherichia coli* strain.

In a study conducted by Gomori et al. [72], it was shown that in the EO of another species of *Thymus*, there is a high production of *p*-cymene, which retains good antimicrobial activity and is enhanced by the combined use of thymol. Thymol ether also has some antimicrobial activity, which is higher on *S. aureus* and lower on *E. coli*. In the literature, this compound is rarely analyzed for its antimicrobial activity, and this is another novelty of this study.

In general, thymol and especially *β*-bisabolene were the single compounds responsible for the antimicrobial activity of *T. richardii* EO [73]. According to Braga [74], thymol has excellent antimicrobial properties; it acts on bacterial and fungal adhesion to various types of eukaryotic cells as well as possessing strong antioxidant activity, for example, protecting the vaginal cells.

### 2.4. Fluorescence Microscopy Analysis

To study the action mechanism of (**F**) and its main compounds that are most involved in its antimicrobial activity, fluorescence microscopy experiments were performed. To verify the effect of EO and single compounds on bacterial membrane integrity, *E. coli* and *S. aureus* cells were used and stained with DAPI, a fluorescent stain for DNA that emits blue light, and propidium iodide, which emits red light.

The latter can enter cells only through damaged membranes and is therefore considered an indicator of cell membrane damage.

**Figure 5:**
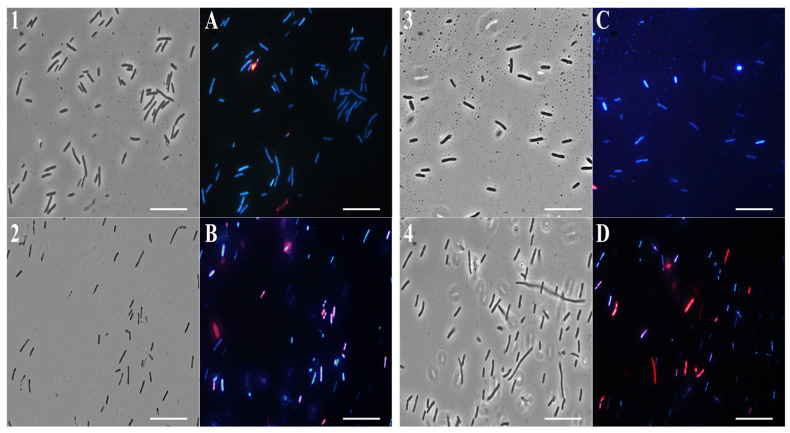
Fluorescence microscopy images. Panels show *E. coli* bacterial cells. Panels (**1**–**4**) obtained from optical microscope images, and (**A**–**D**) from fluorescence microscope images. Untreated bacterial cells (**1**,**A**); cells treated with (**F**) (**2**,**B**); cells treated with *β*-bisabolene (**3**,**C**); cells treated with thymol (**4**,D).

**Figure 6:**
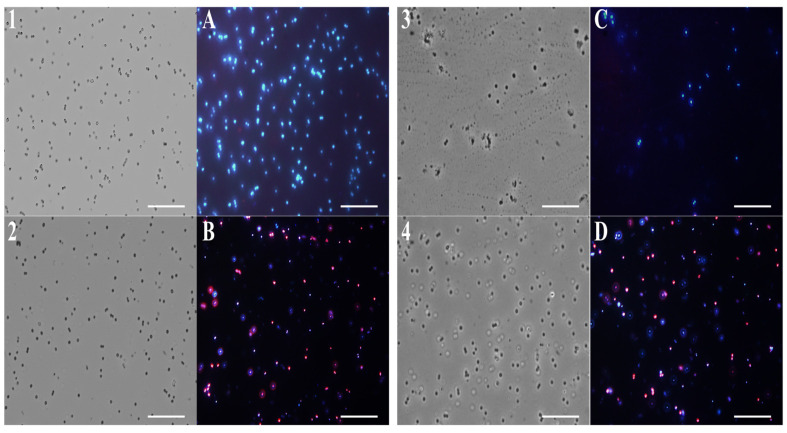
Fluorescence microscopy images. Panels show *S. aureus* bacterial cells. Panels (**1**–**4**) obtained from optical microscope images, and (**A**–**D**) from fluorescence microscope images. Untreated bacterial cells (**1**,**A**); cells treated with (**F**) (**2**,**B**); cells treated with *β*-bisabolene (**3**,**C**); cells treated with thymol (**4**,**D**).

As shown in Figure 5 and Figure 6, panels 1, and A, untreated bacterial cells—used as a control—appear intact and blue because of DAPI fluorescence.

Notably, after 4 h of EO (0.5 mg/mL) treatment, a significant amount of *E. coli* (Figure 5B) and *S. aureus* (Figure 6B) cells developed a red fluorescence, suggesting breakdown of membranes. After the *β*-bisabolene treatment, *E. coli* and *S. aureus* membranes are intact, and bacterial cells appear blue for the entry of DAPI into the bacterial cell (Figure 5C and Figure 6C). As shown in Figure 5 and Figure 6, respectively, in panels D, treatment with thymol at the percentage contained in thyme EO causes damage to the membranes after 4 h of exposure to the compound itself. Results analysis suggests that EO biocide action towards Gram-negative and -positive strains might likely be exerted through membrane damage, in accordance with previous reported studies [75].

In fact, it is known in the literature that monoterpenes damage the biomembranes of both Gram-positive and Gram-negative bacteria. These compounds disturb the lipid fraction of the microorganism’s plasma membrane, causing alterations in the permeability and leakage of intracellular material [76]. This effect may be related to the physicochemical characteristics of the EO, the lipid composition, and the net surface charge of the microbial membranes.

*T. richardii* EO probably exerts its antimicrobial action through thymol. Its chemical structure is hydrophobic, which suggests a capacity to permeabilize the cell membrane. Several reports exploring the action mechanisms of phenolic compounds have indicated that they mainly disrupt bacterial cell membranes, resulting in a leakage of intracellular materials required for normal metabolism and survival directed against bacterial membranes [77]. The damage to the membrane probably favors the entry of *β*-bisabolene (which belongs to the polygodial class) into the bacterial cells.

Although these authors also showed that this class of compounds inhibited both respiration and the synthesis of cellular macromolecules, such as DNA, RNA, proteins, and polysaccharides, they concluded that these were secondary effects of the cell damage caused by polygodial since the inhibition of these macromolecules was not specific [78]. Probably, the simultaneous action of all the compounds contained in the EO is essential to exerting the antimicrobial activity.

### 2.5. Antibiofilm Activity of Essential oils (F), Thymol, and β-Bisabolene

Essential oil (**F**) used in low concentrations may have properties that prevent the formation of bacterial biofilms. As it is known from previous studies, different EOs [79], even at low concentrations, can have an antibiofilm effect. To validate this hypothesis, experiments on a biofilm-forming model strain (*M. smegmatis*) were performed.

**Figure 7:**
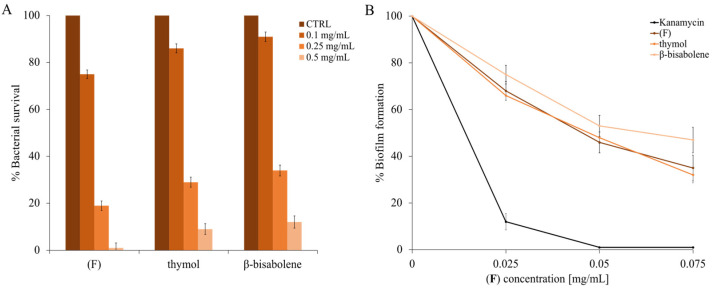
Determination of the antibiofilm activity. Panel (**A**) shows the percentage of antimicrobial activity determined by (**F**), *β*-bisabolene and thymol against *S. smegmatis*. Panel (**B**) shows the percentage of *M. smegmatis* biofilm formation. Different concentrations of (**F**), *β*-bisabolene and thymol were tested (x-axis). DMSO is the negative control, and kanamycin is the positive control. The assays were performed in three independent experiments. Standard deviations are always less than 5%.

The dose-response curves are shown in Figure 7A, and MIC values were also calculated to identify the concentrations of thymol, *β*-bisabolene, and EO that did not cause the bacterium’s death. Once the concentration that did not inhibit the growth of *M. smegmatis* was identified, lower concentrations were used, from 0.01 to 0.075 mg/mL (Figure 7B). As can be seen in panel B of the same figure (Figure 7), there is a biofilm inhibition of about 60% using both the (**F**) and thymol compounds, while the *β*-bisabolene percentage is slightly smaller (about 50%).

This aspect has a significant impact on the possible use of EO to preserve plants from pathogenic bacteria [80]. Indeed, the failure of conventional antibiotic treatments suggests that the eradication of microbial biofilms needs continuous updating [81]. Natural anti-biofilm substances target persistent biofilms and promote the diffusion of antimicrobials in the biofilm matrix. Usually, these natural agents are active at different stages of biofilm formation to degenerate the matrix and eventually kill the released cells. The goal of an antibiofilm agent is to destroy the biofilm and kill the bacterial cells contained in it; for this purpose, our thymus EO could be employed.

There are many new applications of thyme EO; for example, in a study by Arrais et al. [82], the inclusion of the EO in tablets allows a gradual and prolonged release, increasing the exposure time of the bacteria to the latter. This application is especially ideal for microbial biofilms of *S. aureus* and *P. aeruginosa* that are more difficult to eradicate than planktonic bacterial cells.

### 2.6. Cytotoxic Activity of (F) and Its Principal Components

**Figure 8:**
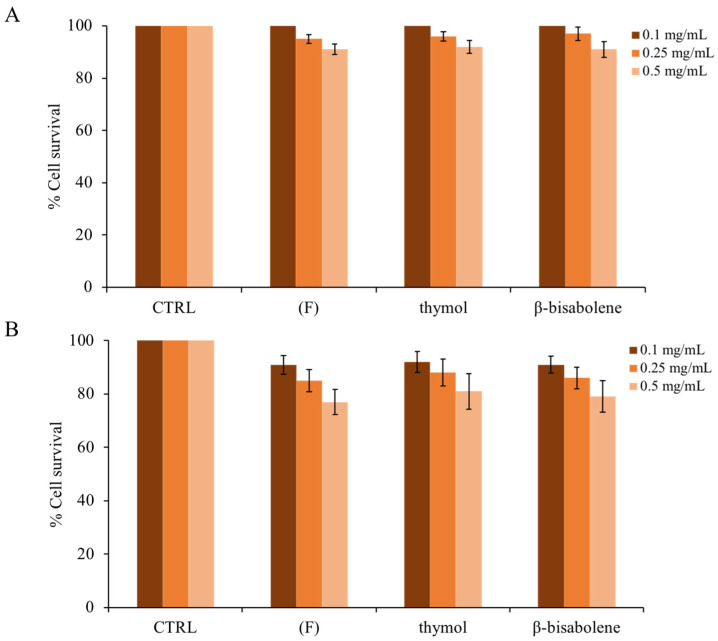
Cytotoxic effect of (**F**) and of the individual components present on eukaryotic cells. HaCat cells were treated for 4 h (**A**) and for 24 h (**B**). Cellular cytotoxicity was determined by the MTT assay. The assays were performed in three independent experiments. Standard deviations are always less than 5%.

To verify whether the EO of thyme and the compounds found within it could be toxic to eukaryotic cells, human keratinocytes were used to perform an assay using the MTT reagent, as reported in the methods. As shown in Figure 8A (4 h exposure to compounds), even at the maximum concentration (0.5 mg/mL), the compounds are not cytotoxic. Increasing the exposure time to 24 h at the maximum concentration (panel B), the compounds begin to exert a slight cytotoxic effect [49]. Thus, it is possible to conclude that under the experimental conditions used, (**F**) is non-toxic to this cell line. Similar results were observed in a study in which the EO of the thymus presented antimicrobial activity against several microorganisms, including *Pseudomonas aeruginosa*, *Proteus vulgaris*, *Citrobacter koseri*, and *Klebsiella pneumoniae* [83]. According to our observations, the EO of thyme does not affect HaCaT cell viability.

### 2.7. Antioxidant Activity of T. richardii EO (F)

With regard to the antioxidant activity, the main action was shown by the extracts, like that of *T. laevigatus* Vahl. (strong radical scavenging activity in the DPPH assay compared to the standard antioxidant) and *T. vulgaris* [dose-dependent DPPH-scavenging capability similar to standard antioxidants butylhydroxyanisol (BHA) and butylated hydroxytoluene (BHT)] [60,84]. The essential oil from *T x citriodorus* (Pers.) Schreb. leaves had relevant cytotoxic activity against HepG2 cells, inducing apoptosis with the expression of NF-*κ*B [85]. Essential oils of Greek *T. vulgaris* instead attenuated the LPS-induced elevation in nuclear factor-kappa (NF-*κ*B), cyclooxygenase-2 (COX-2), TNF-g, inducible nitric oxide synthase (iNOS), NO, and oxidative stress [86].

**Figure 9:**
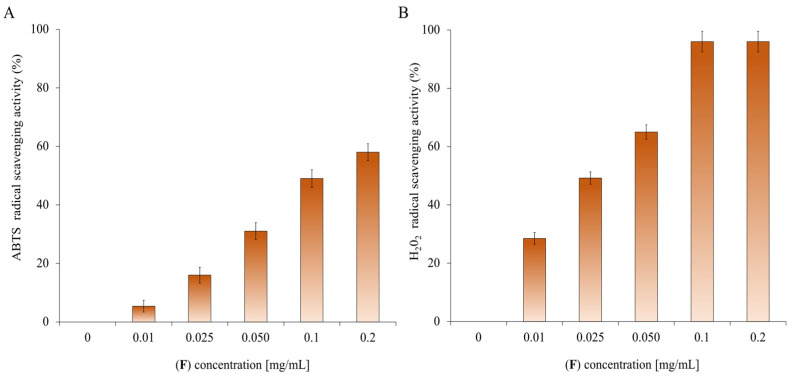
Determination of the antioxidant activity of (**F**). Panel (**A**) shows the abatement activity of ABTS radicals reported as % of ABTS removed with respect to the control. Panel (**B**) shows the hydrogen peroxide scavenging activity reported as % of H_2_O_2_ removed relative to the control. The data are the mean of three independent experiments. Standard deviations are always less than 10%.

EO (**F**) is rich in oxygenated monoterpenes; these types of compounds possess various biological properties, including antioxidant ones [87]. Other studies on the antioxidant activity of EO have shown that the abatement ability of ABTS radicals is closely related to the concentration of EOs and has a strong connection with its chemical components, especially its main constituents [88]. The primary components of (**F**) are oxygenated terpenes, which have a great impact on the antioxidant activity of the EO. According to the analysis of the primary components of the EO, the antioxidant activity is positively correlated with the amount of oxygenated terpenoids (oxygenated monoterpenes and sesquiterpenes) [89,90]. Figure 9 shows the increasing percentage of scavenging activities of ABTS and H_2_O_2_ radicals as the concentration (0–0.2 mg/mL) of EO increases. The data shown in Figure 9 are expressed in Table 3 and Table 4 as IC_50_ values, representing the EO concentration that causes a 50% reduction in ABTS (Table 3) and H_2_O_2_ (Table 4) radicals. The EO shows anti-H_2_O_2_ activity with IC_50_ values of 0.2 mg/mL and the lowest anti-radical effect (IC_50_ value > 100 mg/mL) for ABTS.

**Figure 10:**
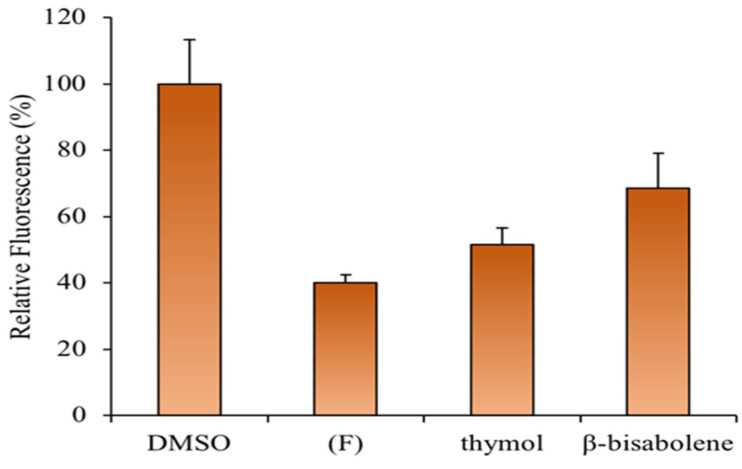
Effects of (**F**), thymol, and *β*-bisabolene on intracellular ROS generation in HaCaT cells upon H_2_O_2_-treatment.

ROS-sensitive fluorescent dye was used to investigate whether the (**F**) prevents H_2_O_2_-induced ROS generation. HaCaT cells that had been exposed to H_2_O_2_ showed a significant increase in the accumulation of intracellular ROS, whereas this induction was significantly inhibited by the EO or thymol pretreatment (Figure 10). Accordingly, recent studies revealed an increased antioxidant effect of *Thymus* EOs in HaCat cells in a dose-dependent manner, as observed in this study [83].

## 3. Materials and Methods

### 3.1. Plant Material

The pre-flowering aerial parts of *T. richardii* subsp. *nitidus* were collected at Punta Madonnuzza on Marettimo Island, Sicily, Italy (37°59′03″ N, 12°03′06″ E, 400 m a.s.l.), in April 2022, and a voucher specimen has been deposited in the STEBICEF Department, University of Palermo (PAL113474). The full flowering material was collected in the same location in June 2022.

### 3.2. Isolation of Essential Oil

The extraction of EOs was carried out according to Basile et al. [91]. Fresh samples were ground in a Waring blender and then subjected to hydrodistillation for 3 h, according to the standard procedure described in the European Pharmacopoeia (2020). The EO_S_ were dried over anhydrous sodium sulfate and stored in sealed vials under N_2_ at −20 °C, ready for the GC-MS and GC-FID analyses; the samples yielded 0.05% and 0.07% of oils (*w*/*w*) for PF and F, respectively.

### 3.3. GC-MS Analysis

The analysis of EOs was performed according to the procedure reported by Badalamenti et al. [92]. GC-MS analysis was performed using a Shimadzu QP 2010 plus equipped with an AOC-20i autoinjector (Shimadzu, Kyoto, Japan) gas chromatograph equipped with a FID, a capillary column (DB-Wax) 30 m × 0.25 mm i.d., film thickness 0.25 μm, and a data processor. The oven program was as follows: temperature increase at 40 °C for 5 min, at a rate of 2 °C/min up to 260 °C, then isothermal for 20 min. Helium was used as a carrier gas (1 mL min^−1^). The injector and detector temperatures were set at 250 °C and 290 °C, respectively. One μL of EO solution (3% EO/Hexane *v*/*v*) was injected with split mode 1.0; MS range 40–600. The percentages in Table 1 are calculated with the TIC from MS. The settings were as follows: ionization voltage, 70 eV; electron multiplier energy, 2000 V; transfer line temperature, 295 °C; solvent delay, 4 min. Linear retention indices (LRI) were determined by using retention times of *n*-alkanes (C_8_–C_40_), and the peaks were identified by comparison with mass spectra and by comparison of their relative retention indices with WILEY275, NIST 17, ADAMS, and FFNSC2 libraries.

### 3.4. Pure Compounds

Limonene and *β*-bisabolene were purchased from Thermo Fisher Scientific Inc. (Waltham, MA, USA), *p*-cymene and thymol from Tokyo Chemical Industry Co. (Chuo-Ku, Tokyo, Japan), while the thymol methyl ether (54 mg) was prepared from thymol (50 mg) using diazomethane under stirring for 30 min.

### 3.5. Bacterial Strains

Gram-negative bacteria: *Escherichia coli* DH5α, *Pseudomonas aeruginosa* PAOI ATCC 15692, and *Salmonella typhimurium* ATCC14028, and Gram-positive ones: *Staphylococcus aureus* ATCC6538P, Streptococcus oralis CECT 8313, *Streptococcus mutans* ATCC 35668, and *Mycobacterium smegmatis* mc^2^ 155, were chosen to evaluate antibacterial activity.

### 3.6. Antimicrobial Activity Assay

The presence of antimicrobial molecules in (F) was detected using the Kirby-Bauer test with modifications [93]. Three volumes (10, 15, and 20 µL) of EO [5 mg/mL] were placed on Luria bertani agar plates and then coated with the indicator strains: *E. coli* and *S. aureus*. The negative control was dimethyl sulfoxide (15 µL) used to resuspend (F); the positive control was the antibiotic ampicillin (1 µL) concentrated at 5 mg/mL. Antimicrobial activity was calculated as reported in Pota et al. [94].

Another method to evaluate the antimicrobial activity involved the Gram-positive and Gram-negative strains cell viability counting. Microbial cells were treated with both EOs at 0.10, 0.25, and 0.50 mg/mL concentrations. Microbial cells without EOs were the positive control; instead, cells with DMSO were used as the negative control. The following day, the survival rate of bacterial cells was calculated by counting the colonies [95]. The same assay was carried out to evaluate the antimicrobial activity of compounds mostly present in (**F**). Each compound was tested at its EO maximum concentration, considering the percentage at which it is present in the EO. In this study, the main constituents of the EO collected at the full flowering stage were *p*-cymene ~9% (0.045 mg/mL), *β*-bisabolene ~18% (0.09 mg/mL), limonene ~16% (0.08 mg/mL), thymol methyl ether ~9% (0.045 mg/mL, and thymol ~16% (0.08 mg/mL). All experiments were carried out in triplicate, and the reported result was an average of three independent experiments (*p* value of < 0.05).

### 3.7. Determination of Minimal Inhibitory Concentration

Minimal Inhibitory Concentrations (MICs) of (F) against the Gram-positive and Gram-negative strains were determined according to the microdilution method established by the Clinical and Laboratory Standards Institute (CLSI) [96]. Five samples of 10^5^ CFU/mL were added to 95 µL of Mueller-Hinton broth (CAM-HB; Difco), supplemented or not with various concentrations (0.1–0.5 mg/mL) of (F). After overnight incubation at 37 °C, MIC_100_ values were determined to be the lowest concentration responsible for the lack of bacterial growth.

### 3.8. Fluorescence Microscopy Experiments

*E. coli* DH5α and *S. aureus* ATCC6538P cells were incubated in the dark for 4 h at 37 °C with or without (F) 0.5 mg/mL, *β*-bisabolene 0.09 mg/mL, and thymol 0.08 mg/mL. Samples were observed as described in Di Napoli et al. [97].

### 3.9. Antibiofilm Inhibition Tests

The antibiofilm activity against *M. smegmatis* mc^2^ 155 was evaluated by colorimetric testing. Microbial cells with DMSO were the negative control, and the antibiotic kanamycin (2 µg/mL) was the positive control. Treated prokaryotic cells contained EO (0.025, 0.05, and 0.075 mg/mL), 16% thymol, and 18% *β*-bisabolene at these EO concentrations. The plate was incubated at 37 °C for 36 h [79]. The percentage of biofilm formed was evaluated by comparing the optical density values of the treated and untreated samples.

### 3.10. Eukaryotic Cell Culture

HaCat cells (human keratinocytes) are immortal keratinocyte cell lines used in research laboratories [98]. These cells were grown in Dulbecco’s Modified Eagle Medium (DMEM) at 37 °C with 5% CO_2_. EO (F), *β*-bisabolene, and thymol at concentrations of 0.10, 0.25, and 0.50 mg/mL, respectively, were used for the MTT assay [99].

### 3.11. ABTS and H_2_O_2_ Scavenging Capacity Assay

For this test, based on the scavenging of ABTS radicals, the protocol of Napolitano et al. was used [100]. The ABTS solution was added to 100 µL of EO and/or individual compounds (concentrations of 0.01, 0.025, 0.05, 0.1, and 0.2 mg/mL). Each test was performed at least three times.

The scavenging capacity of H_2_O_2_ was evaluated by the variation of the absorbance at 240 nm, as described in the literature [101]. Different concentrations (0.01, 0.025, 0.05, 0.1, and 0.2 mg/mL) of EO and individual compounds were mixed with the hydrogen peroxide. After half an hour, the concentration of hydrogen peroxide was calculated by measuring the absorbance. Each assay was performed at least three times.

### 3.12. Antioxidant Test on HaCat Cells

HaCaT cells were seeded in 12-well plates and then incubated at 37 °C with 5% CO_2_ for 24 h. Cells were treated with (F) (0.25 mg/mL), thymol (0.04 mg/mL), *β*-bisabolene (0.045 mg/mL), or DMSO as a control. After 1 h of treatment, cells were exposed to H_2_O_2_ (800 µM) for the next 3 h before intracellular ROS detection. ROS Assay Stain (88-5930, Invitrogen, Waltham, MA, USA) was added to cells in culture media ccording to the manufacturer protocol [102]. Cells were incubated for 1 h at 37 °C with 5% CO_2_. Fluorescence intensity (530 nm) was measured using a Synergy H4 Hybrid Microplate reader (Agilent, Santa Clara, CA, USA).

## 4. Conclusions

This study highlights the antimicrobial properties of *Thymus richardii* subsp. *nitidus* EO, which are especially active against Gram-positive bacteria, pathogens of the oral cavity. Very interesting are the properties of thymol and *β*-bisabolene, which become the major constituents of the EO antimicrobial activity; they have good activity at much lower concentrations than 0.5 mg/mL. Furthermore, the EO of this *Thymus* species inhibits the formation of model biofilms at low concentrations—nearly 50% at 0.75 mg/mL. The beneficial effect it has on eukaryotic cells is demonstrated by its low toxicity and the antioxidant action it is able to exert on human epithelial cells. The most common dental diseases are dental cavities, periodontitis, gingivitis, and oral cancer. EOs seem to have a beneficial role in each one of them. This study is based on an endemic Sicilian species of *Thymus*; the composition of its essential oil is very interesting and differs from many other species due to the abundant presence of *β*-bisabolene. Furthermore, the latter, together with thymol, constitutes a compound with high antimicrobial activity that is essential for the correct functioning of thyme as an antibacterial agent. This new oil has multiple properties and can potentially be used in various fields, ranging from food preservation to cosmetics or even in dentistry. In this case, *Thymus richardii* subsp. *nitidus* EO represents a valid ally for oral healthiness.

## Figures and Tables

**Table 1:** Chemical composition (%) of *Thymus richardii* subsp. *nitidus* EO before and during flowering, collected in Sicily, Italy.

No.	Compounds ^a^	LRI ^b^	LRI ^c^	Pre-Flowering (%)	Flowering (%)
1	*α*-Pinene	1009	1017	1.40	1.28
2	3-Thujene	1029	1035	-	0.59
3	*β*-Pinene	1094	1099	-	0.11
4	Sabinene	1103	1115	-	0.13
5	3-Carene	1135	1143	-	0.05
6	4-Carene	1148	1157	0.50	1.32
7	Limonene	1185	1193	6.23	15.59
8	Sylvestrene	1200	1205	-	1.16
9	*γ*-Terpinene	1237	1255	1.34	-
10	*p*-Cymene	1256	1272	24.45	9.04
11	*α*-Copaene	1492	1497	-	0.10
12	*α-*Bourbonene	1523	1528	-	0.39
13	Linalool	1546	1553	0.18	6.70
14	Thymol methyl ether	1595	1604	15.90	8.87
15	Dihydrocarvone	1618	1624	5.04	-
16	*α*-Amorphene	1666	1675	0.74	0.48
17	*γ-*Muurolene	1700	1704	0.39	0.54
18	Germacrene D	1701	1706	3.19	6.14
19	*β*-Bisabolene	1739	1741	28.54	17.91–
20	*cis*-*α*-Bisabolene	1748	1759	0.90	-
21	Thymol	2184	2198	4.56	16.26
22	Carvacrol	2231	2239	4.49	7.82
23	*τ*-Cadinol	2180	2187	-	2.13
24	*α*-Cadinol	2248	2255	0.28	1.01
	Monoterpene Hydrocarbons			33.92	29.27
	Oxygenated Monoterpenes			30.17	39.65
	Sesquiterpene Hydrocarbons			33.76	25.56
	Oxygenated Sesquiterpenes			0.28	3.14
	Total			98.13	97.62

^a^ Components listed in order of elution on an DB-Wax column; ^b^ Linear retention index on a DB-Wax polar column; ^c^ Linear retention indices based on literature (https://webbook.nist.gov/).

**Table 2:** Determination of minimum concentration values (MIC) inhibiting bacterial growth. The MIC_100_ is expressed in mg/mL of (**F**) against Gram negative and Gram-positive bacteria. The values were obtained from a minimum of three independent experiments.

Strains	MIC_100_ [mg/mL]
*E. coli* DH5α	>0.5
*P. aeruginosa* PAO1	>0.5
*S.* Typhimurium ATCC14028	>0.5
*S. aureus* ATCC6538P	0.5
*S. mutans* ATCC 35668	0.5
*S. oralis* CECT 8313	0.25

**Table 3:** Concentration at 50% scavenging activity. ABTS: 2,20-azino-bis (3-ethyl-benzothiazoline-6-sulfonic acid); H_2_O_2_: hydrogen peroxide. The positive control is ascorbic acid for ABTS and resveratrol for H_2_O_2_.

Sample	IC_50_ of ABTS (mg/mL)	Sample	IC_50_ of H_2_O_2_ (mg/mL)
(**F**)	0.1	(**F**)	0.025
Ascorbic acid	0.00003	Resveratrol	0.00005

**Table 4:** Concentration at 50% scavenging activity. ABTS: 2,20-azino-bis (3-ethyl-benzothiazoline-6-sulfonic acid); H_2_O_2_: hydrogen peroxide. The positive control is ascorbic acid for ABTS and resveratrol for H_2_O_2_.

Sample	IC_50_ of H_2_O_2_ (mg/mL)	IC_50_ of ABTS (mg/mL)
*p*-Cymene	0.27	0.7
Thymol methyl ether	0.45	0.1
Limonene	0.25	1
Thymol	0.15	<0.1
*β*-Bisabolene	0.25	0.25
CTRL	0.00005	0.00003

## Data Availability

Not applicable.

## Sample Availability

Samples of the compounds are available from the authors.
